# iMEC: Online Marker Efficiency Calculator

**DOI:** 10.1002/aps3.1159

**Published:** 2018-06-24

**Authors:** Ali Amiryousefi, Jaakko Hyvönen, Péter Poczai

**Affiliations:** ^1^ Organismal Evolutionary Biology Research Program Faculty of Biology and Environmental Sciences Viikki Plant Science Centre University of Helsinki P.O. Box 65 Helsinki FI‐00014 Finland; ^2^ Botany Unit Finnish Museum of Natural History University of Helsinki P.O. Box 7 Helsinki FI‐00014 Finland

**Keywords:** arbitrarily amplified dominant markers (AADs), DNA band, molecular marker, multi‐locus fingerprinting, polymorphism

## Abstract

**Premise of the Study:**

To accurately design plant genetic studies, the information content of utilized markers and primers must be calculated. Plant genotyping studies should take into account the efficiency of each marker system by calculating different parameters to find the optimal combination of primers. This can be problematic because there are currently no easily accessible applications that can be used to calculate multiple indices together.

**Methods and Results:**

The program Online Marker Efficiency Calculator (iMEC) was developed using R for the simple computation of seven polymorphism indices (heterozygosity index, polymorphism information content, discriminating power, effective multiplex ratio, marker index, arithmetic mean heterozygosity, and resolving power). These indices are based on dominant and codominant DNA fingerprinting markers, thus allowing comparison and selection of optimal genetic markers for a given data set.

**Conclusions:**

iMEC simplifies the calculation of diverse indices for the marker of choice to better enable researchers to measure polymorphism information for individual markers. The program is available at https://irscope.shinyapps.io/iMEC/.

Molecular markers are applied across numerous scientific fields from developmental biology, systematics, and conservation biology to forensic studies (Schlötterer, 2004). They play a pivotal role in constructing genetic maps and identifying individuals with certain genes, as well as for studying genetic variability. In plant sciences, molecular tools have become key to identifying species and determining relationships for plant production and supervision of intellectual property rights. Determining genetic relationships is essential for evolutionary and conservation studies, as well as in the selection of germplasm for plant breeding. The persistent need for the continuous development of genetically improved crops to satisfy the demands of the increasing human population is strongly dependent on the development of various molecular markers (Henry, 2012).

Molecular marker technologies have evolved from the use of isozymes to hybridization‐based DNA methods. With the development of PCR, these techniques were replaced by arbitrarily amplified dominant (AAD) markers (e.g., amplified fragment length polymorphism [AFLP], inter‐simple sequence repeat [ISSR], and random‐amplified polymorphic DNA [RAPD] markers) and microsatellites (simple sequence repeats [SSRs]). The rapid development of public genomic databases subsequently initiated a trend to abandon AAD markers for functional markers (Poczai et al., 2013). This latter type of markers, such as conserved DNA‐derived polymorphism (CDDP) and intron‐targeting (IT) markers, are superior to randomly generated markers because they are gene‐targeted and derived from sequences affecting phenotypic variation. Recent advances that have lowered the cost of high‐throughput sequencing technology have led to the development of genotyping using next‐generation sequencing (Miller et al., 2007; Elshire et al., 2011; Vartia et al., 2016). These developments have significantly changed the approach to marker discovery and analyses.

The choice of molecular markers largely depends on the level of polymorphism to be detected and their genomic coverage, rather than on the technology used to generate the markers. Estimates of marker‐based selection depend on the linkage of the genomic region and the marker itself. Because highly informative markers can reduce the amount of genotyping required for inference of ancestry, it is desirable to measure the extent to which specific markers contribute to this inference (Rosenberg et al., 2003). Several approaches have been previously developed for measuring polymorphism information (Table 1), but a user‐friendly platform to calculate this information is missing or otherwise inaccessible (see PICcalc; Nagy et al., 2012). Here, we introduce the program Online Marker Efficiency Calculator (iMEC), an online calculator for deriving polymorphism statistics of individual molecular markers.

**Table 1:** Detailed description of polymorphism indices calculated by iMEC

Index	Formula	Definition
Expected heterozygosity^a^	*H* = 1 – Σ *p*_i_^2^	The probability that an individual is heterozygous for the locus in the population. *p*_i_ is the allele frequency for the *i*‐th allele, and the summation is over all available alleles.
Polymorphism information content^b^	*PIC* = 1 – Σ *p*_i_^2^ – Σ Σ *p*_i_^2^ *p*_j_^2^	The probability that the marker genotype of a given offspring will allow deduction, in the absence of crossing over, of which of the two marker alleles of the affected parents it received. *p*_i_ and *p*_j_ are the population frequency of the *i*‐th and *j*‐th allele. The first summation is over the total number of alleles, whereas the two subsequent summations denote all the *i* and *j* where *i* ≠ *j*.
Effective multiplex ratio^c^	*E* = *n β*	The product of the fraction of polymorphic loci for an individual assay. In other words, the number of loci polymorphic in the germplasm set of interest analyzed per experiment fraction of polymorphic loci. Defining *β = n*_p_ / (*n*_p_ + *n*_np_), where *p* and *np* indicate the polymorphic and nonpolymorphic fraction of the markers, so *n*_p_ and *n*_np_ represent their respective counting numbers.
Mean heterozygosity^c^	*H*_avp_ = Σ *H*_n_ / *n*_p_	The average heterozygosity calculated for polymorphic markers. *H*_n_ is the heterozygosity of the polymorphic fraction of markers, and the summation is over all of the polymorphic loci *n*_p_.
Marker index^c^	*MI* = *E H*_avp_	The product of the effective multiplex ratio and the average expected heterozygosity for polymorphic markers, where *H*_avp_ denotes the average expected heterozygosity for the polymorphic markers. It is equal to Σ *H*_p_ / *n*_p_, where the summation is over all polymorphic sites with *H*_p_ and *n*_p_ defined as above.
Discriminating power^d^	*D* = 1 – *C*	The probability that two randomly chosen individuals exhibit different banding patterns and are thus distinguishable from one another. *C* is defined as the confusion probability. For the *i‐*th pattern of the given *j‐*th primer, present at frequency *p*_i_ in a set of varieties, the confusion probability is *C* = Σ *c*_i_ = Σ *p*_i_ $\frac{Npi-1}{N-1}$ where for *N* individuals, *C* is equal to the sum of all *c*_i_ for all of the patterns generated by the primer.
Resolving power^e^	*R* = Σ *I*_*b*_	Resolving power is based on the distribution of alleles within the sampled genotypes and strongly correlates with the ability to distinguish between analyzed samples. The division of samples into two groups is based on the presence or absence of a band, ideally present in one part of the samples while absent from the other. Bands can be weighed according to their similarity to the optimal condition (50% of genotypes containing the band), where *I*_b_ or band informativeness is represented on a scale of 0–1 and is defined as *I*_b_ = 1 – (2 × |0.5 – *p*|), where *p* is the portion of the samples containing the observed band. Using this value, the resolving power or the ability of a primer (technique) to distinguish between genotypes could be represented by the sum of these adjusted values for all generated bands.

a Liu (1998)

b Botstein et al. (1980)

c Powell et al. (1996)

d Tessier et al. (1999)

e Prevost and Wilkinson (1999)

## METHODS AND RESULTS

iMEC is coded in R and is available as a Web application at https://irscope.shinyapps.io/iMEC/. The software can be used online or, alternately, users can access and modify the source code deposited on GitHub (https://github.com/Limpfrog/iMEC). For more advanced users of R, this option allows for more versatile use of the program. In addition, the test data used for benchmarking the software are also available online and can be used as example files to run the program. The software reads standard PHYLIP (.phy) (Felsenstein, 2002) and NEXUS (.nex) (Maddison et al., 1997) file formats, which are widely supported by other software and can be easily created using a text editor or other programs (e.g., NEXUS Data Editor [Page, 2001] and Mesquite [Maddison and Maddison, 2018]). iMEC is able to handle diverse types of data including DNA generated by high‐throughput sequencing, microsatellites, and AADs such as AFLP markers. Input data must be binary coded (0, 1) or recorded as multi‐state characters (0, 1, 3, etc.). For example, AAD markers should be recorded in presence/absence matrices, whereas microsatellite and single‐nucleotide polymorphism data sets can be scored either in binary or in multi‐state format. As basic measures, iMEC calculates heterozygosity index (*H*), polymorphism information content (*PIC*), discriminating power (*D*), effective multiplex ratio (*E*), marker index (*MI*), arithmetic mean heterozygosity (*H*_avp_), and resolving power (*R*) (Table 1). It is important to note that, for AAD markers, iMEC presumes that fragments of equal length amplify from the corresponding loci and that they represent a single, dominant locus with two possible alleles (presence/absence). Therefore, patterns generated by AAD markers represent multiple loci, whereas it is assumed that SSRs or similar codominant systems reveal multiple alleles of a single locus, which is not always the case. The occurrence of non‐homologous fragments of the same size (size homoplasy) is a constraint of SSRs, which is caused by insertion/deletion polymorphisms (indels) in microsatellite flanking regions. For codominant markers, the program assumes that each assay reveals a single locus and assigns an *E* value of 1 for each marker. Table 1 summarizes these seven calculative indices with their respective details.

We ran iMEC on an example data set taken from Poczai et al. (2011) using CDDP and IT markers on a germplasm set of bittersweet (*Solanum dulcamara* L.), consisting of 96 accessions. The data set is available for download, together with other example files, from the application's website, and the resulting calculations are summarized in Table 2. The maximum value of *H* and *PIC* for binary data is 0.5, because two alleles per locus are assumed, and both are influenced by the number and frequency of the alleles; for codominant markers, these values vary between 0 and 1. In the example data, high values indicate the advanced discriminatory capacity of both marker systems. A closer inspection of the *MI* generated for the two different assays highlights the distinguishing power of CDDP markers compared to IT markers, which is due to a higher effective multiplex ratio component. *R* provided the basis for comparing the diagnostic effectiveness of primers used in the bittersweet example. The combined *R* value of the primers also provides a measure of their collective performance for identification purposes. The primer MADS‐A alone could identify 53 bittersweet genotypes, according to the equation of Prevost and Wilkinson (1999; 0.15*x* + 1.78 = *R*, where *x* is the number of genotypes identified). The combination of two CDDP primers (MADS‐A and WRKY‐B) or one CDDP primer together with one IT primer of the highest *R* value (e.g., GPSS‐943 or S2‐317) can identify all of the bittersweet accessions (*x* > 100). For future germplasm management and genetic diversity assessment, these markers are the most ideal choices. Comparison of the average *R* value of IT and CDDP markers also reveals that the latter performs better in identification of accessions.

**Table 2:** Polymorphism statistics calculated with iMEC for different types of primers for the bittersweet (*Solanum dulcamara*) data set

Primer name	Scored bands	*H*	*PIC*	*E*	*H*_avp_	*MI*	*D*	*R*
**CDDP primers**								
WRKY‐A	10	0.4672	0.3906	6.2813	0.0005	3.8030	0.6057	4.7708
WRKY‐B	12	0.4230	0.4103	3.6458	0.0004	3.3093	0.9079	7.0833
MYB	9	0.4998	0.3748	4.5938	0.0006	3.3970	0.7398	6.0625
ERF	12	0.4415	0.4023	3.9479	0.0004	3.5206	0.8920	7.1042
KNOX	10	0.4639	0.3921	6.3438	0.0005	3.7908	0.5978	5.3125
MADS‐A	15	0.4979	0.3758	7.9896	0.0003	5.7229	0.7165	9.8125
MADS‐B	12	0.4614	0.3933	4.3333	0.0004	3.7683	0.8698	5.6250
ABP1‐2	9	0.4869	0.3812	5.2292	0.0006	3.4639	0.6627	5.0833
ABP1‐3	10	0.4792	0.3849	6.0208	0.0005	3.8383	0.6377	5.9167
Average		0.4690	0.3895	5.3762	0.0005	3.8460	0.7366	6.3079
**IT primers**								
Adk‐242	4	0.4043	0.4180	1.1250	0.0011	1.0360	0.9214	2.2500
Adk‐795	4	0.4688	0.3899	1.5000	0.0012	1.2891	0.8600	2.9583
Cat‐232	2	0.2188	0.4758	0.2500	0.0011	0.2461	0.9849	0.5000
Cat‐260	3	0.3680	0.4320	0.7292	0.0013	0.6861	0.9416	1.4167
GPSS‐275	3	0.4946	0.3774	1.3438	0.0017	1.0742	0.8002	1.7708
GPSS‐943	7	0.4330	0.4060	4.7813	0.0006	2.5506	0.5338	3.9792
INHWI‐509	2	0.4980	0.3757	0.9375	0.0026	0.7315	0.7816	0.3750
INHWI‐545	4	0.4761	0.3864	2.4375	0.0012	1.5324	0.6293	2.7917
InG‐220	3	0.4797	0.3847	1.8021	0.0017	1.1518	0.6400	1.7708
LBr‐G9	3	0.4930	0.3782	1.3229	0.0017	1.0657	0.8064	1.8542
S2‐317	6	0.4988	0.3753	2.8542	0.0009	2.2083	0.7741	4.2500
Poni1a‐718	4	0.4066	0.4171	2.8646	0.0011	1.3954	0.4877	0.4375
Average		0.4366	0.4014	1.8290	0.0013	1.2473	0.7634	2.0295

Note

*D* = discriminating power; *E* = effective multiplex ratio; *H* = expected heterozygosity; *H*_avp_ = mean heterozygosity; *MI* = marker index; *PIC* = polymorphism information content; *R* = resolving power.

The *D* parameter described by Tessier et al. (1999) evaluates the efficiency of the primers in identification of bittersweet accessions. In our example, the *D* parameter describes the probability that two randomly chosen bittersweet individuals have different patterns. A higher *D* parameter (closest to 1) implies a lower probability of confusion between bittersweet accessions. For example, *D* parameters of 0.9214 (IT, Adk‐242) and 0.6057 (CDDP, WRKY‐A) are considered highly and moderately polymorphic, respectively. The informativeness of a given marker may differ between collections originating from different regions, as allele frequencies vary between gene pools (Sefc et al., 2000). However, a marker set containing the most informative markers defined in one germplasm collection with high *D* values will also yield high discriminatory power in other gene pools (Sefc et al., 2000). The *D* parameter can also be used to compare different types of marker systems by calculating the average *D* for each class. IT (*D* = 0.7634) and CDDP (*D* = 0.7366) markers have almost equal values, indicating that the two techniques have similar efficiency to discriminate between the accessions. This seems to contradict the interpretation of *R* values, which indicate that CDDPs outperform IT markers. Instead, they show that fewer CDDP primers successfully distinguished among the germplasm set and that the use of additional primers did not increase the overall performance of the marker system. In the case of IT markers, more primer combinations are needed to reach the same efficiency. The addition of more CDDP primers should be avoided and further analysis should be supplemented with IT primers with high *D* values to increase the efficiency of distinguishing among bittersweet accessions.

## CONCLUSIONS

There is currently a wide variety of software tools available for population genetic analyses with dominant markers; these tools feature a number of functions and provide computational possibilities for diverse genetic indices (see Excoffier and Heckel, 2006). However, despite this, no universal application exists that can be used to calculate indices to optimize the choice of molecular markers for plant genetic studies. iMEC software provides a user‐friendly interface to obtain comparative measures for multiplex marker systems. This application will help researchers acquire good estimates of the efficiency of a primer or assay and also allows the comparison of different methods. This software should be of great interest for studies aiming at varietal and species identification using molecular techniques.

## DATA ACCESSIBILITY

The source code used to develop iMEC is available on GitHub (https://github.com/Limpfrog/iMEC). iMEC is available at https://irscope.shinyapps.io/iMEC/.
